# A method for calculating left ventricular ejection fraction noninvasively from left ventricular arterial coupling (Ees/Ea)

**DOI:** 10.1186/s12871-023-02159-0

**Published:** 2023-06-12

**Authors:** Yukiko Yamazaki, Yuka Matsuki, Kenji Shigemi

**Affiliations:** 1 Intensive Care Medicine, University of Fukui Hospital, Fukui, Japan; 2 Department of Anesthesiology and Reanimatology, Faculty of Medicine Sciences, University of Fukui Hospital, 23-3 Eiheijicho, Yoshidagun, 910-1193 Fukui Japan

**Keywords:** Ejection fraction, Left ventricular efficiency, Left ventricular arterial coupling

## Abstract

**Background:**

Ejection fraction (EF), which is assessed using ultrasonography, is a standard parameter for evaluating cardiac function in clinical cardiology and for cardiovascular management during general anesthesia. However, it is impossible to continuously and non-invasively assess EF using ultrasonography. The aim of our study was to develop a method for estimating EF non-invasively using the left ventricular arterial coupling ratio (Ees/Ea).

**Methods:**

Ees/Ea was estimated non-invasively using the parameters pre-ejection period (PEP), ejection time (ET), end-systolic pressure (Pes) and diastolic pressure (Pad), which were calculated from a vascular screening system, VeSera 1000/1500 (Fukuda Denshi Co., Ltd., Tokyo, Japan). Then, left ventricular efficiency (Eff) as a pump, defined as the ratio of external work (EW) to myocardial oxygen consumption, which strongly correlates with the pressure-volume area (PVA), was calculated by a new formula using Ees/Ea, and was used to approximate EF (EFeff). Simultaneously, we measured EF using transthoracic echocardiography (EFecho), and compared it with EFeff.

**Results:**

The study included 44 healthy adults (36 males, 8 females), in whom mean EFecho was 66 ± 5% and EFeff was 57 ± 9%. We found a positive correlation between EFecho and EFeff (R^2^ = 0.219, p < 0.05) on Bland-Altman analysis, with limits of agreement of – 7.5 to 24.4%, and percentage error of 24%.

**Conclusion:**

The results suggest that EF can be measured non-invasively using left ventricular arterial coupling.

## Background

Measurement of ejection fraction (EF) by transthoracic echocardiography (TTE) and transesophageal echocardiography (TEE) is a standard method for evaluating cardiac function. EF and wall motion calculated from TEE are useful during separation from cardiopulmonary bypass and understanding circulatory dynamics during cardiac surgery [1], and for assessment of cardiac function and diagnosis of cardiac ischemia during anesthesia. Perioperative myocardial infarction (PMI) increases the risk of heart failure after coronary artery bypass grafting [2] and postoperative mortality [3]. Early diagnosis and treatment of ischemia is important for anesthesiologists, not only during heart surgery, but also during other procedures. However, cardiac function cannot be calculated continuously from TTE during thoracic surgery, upper abdominal surgery, and with prone positioning. Given this background, a better method of calculating EF noninvasively and continuously is needed.

Left ventricular arterial coupling (Ees/Ea), which is the ratio of end-systolic elastance (Ees) to effective arterial elastance (Ea), expresses the relationship between left ventricular pump function and systemic vascular resistance [4]. In a previous study, Ees/Ea was estimated using a noninvasive method [5].

The aim of our study was to develop a method to estimate EF noninvasively from Ees/Ea.

## Methods

The protocol of this prospective, observational study was approved by the Research Ethics Committee of the University of Fukui Hospital (No. 20,140,124) and the study was conducted in accordance with the Declaration of Helsinki. Informed consent was obtained from all participants and/or their legal guardian(s). Forty-four healthy volunteers were evaluated between 2018 and 2020, after excluding subjects with cardiovascular diseases. Their age, sex, height and weight were recorded.

In all the subjects, transthoracic echocardiography was performed using a Vivid E9 ultrasound (GE Healthcare Japan, Tokyo, Japan) in the left lateral position. EF was obtained by the modified Simpson’s method, and stroke volume (SV) was estimated by subtracting left ventricular end-systolic volume (ESV) from the end-diastolic volume (EDV).

After transthoracic echocardiography, the cardio-ankle vascular index (CAVI) was determined using a vascular screening system, VeSera 1000/1500 (Fukuda Denshi Co., Ltd., Tokyo, Japan) with the participant in the supine position. A standard four-lead ECG and stethoscope were attached to the subject’s chest, and four blood pressure cuffs were attached around the arms and ankles. ECG, phonocardiogram, and brachial arterial pulse waves were continuously recorded. Pre-ejection period (PEP), ejection time (ET), systolic blood pressure (sBP), and diastolic blood pressure (dBP) were automatically recorded.

ET was defined as the interval between the start of the upstroke and the direct notch of the right brachial arterial pulse wave. PEP was calculated by subtracting ET from the time between the Q wave on the ECG and the second heart sound. sBP and dBP were measured using the blood pressure cuffs. End-systolic pressure (Pes) was calculated from sBP and dBP using Kappus et al’s formula [6], as:

1 $${\rm{Pes}}\,{\rm{ = }}\,{\rm{(0}}{\rm{.205}}\, \times \,{\rm{sBP)}}\,{\rm{ + }}\,{\rm{(0}}{\rm{.898}}\, \times \,{\rm{dBP)}}\,{\rm{ + }}\,{\rm{0}}{\rm{.4214}}$$

Additionally, the left ventricular pressure-volume loop, which is a curve used to plot left ventricular volume and left ventricular pressure and is used to understand cardiac mechanics, was plotted using TTE parameters [7]. Left ventricular arterial coupling (Ees/Ea) is the ratio of end-systolic elastance (Ees) to effective arterial elastance (Ea).

Ees represents the slope of the end-systolic pressure volume relationship (ESPVR), and is an index of left ventricular contractile ability [4]. It is calculated using the formula Ees = Pes / (ESV –V0), where V0 is the ventricular volume at zero intraventricular pressure, and Pes and ESV are the end-systolic pressure and volume, respectively. Ea is the slope of the line connecting end-diastolic volume (EDV) on the volume axis to the end-systolic pressure volume point, as an index of afterload. Ea is calculated as the ratio of Pes to stroke volume [4].

**Fig. 1:**
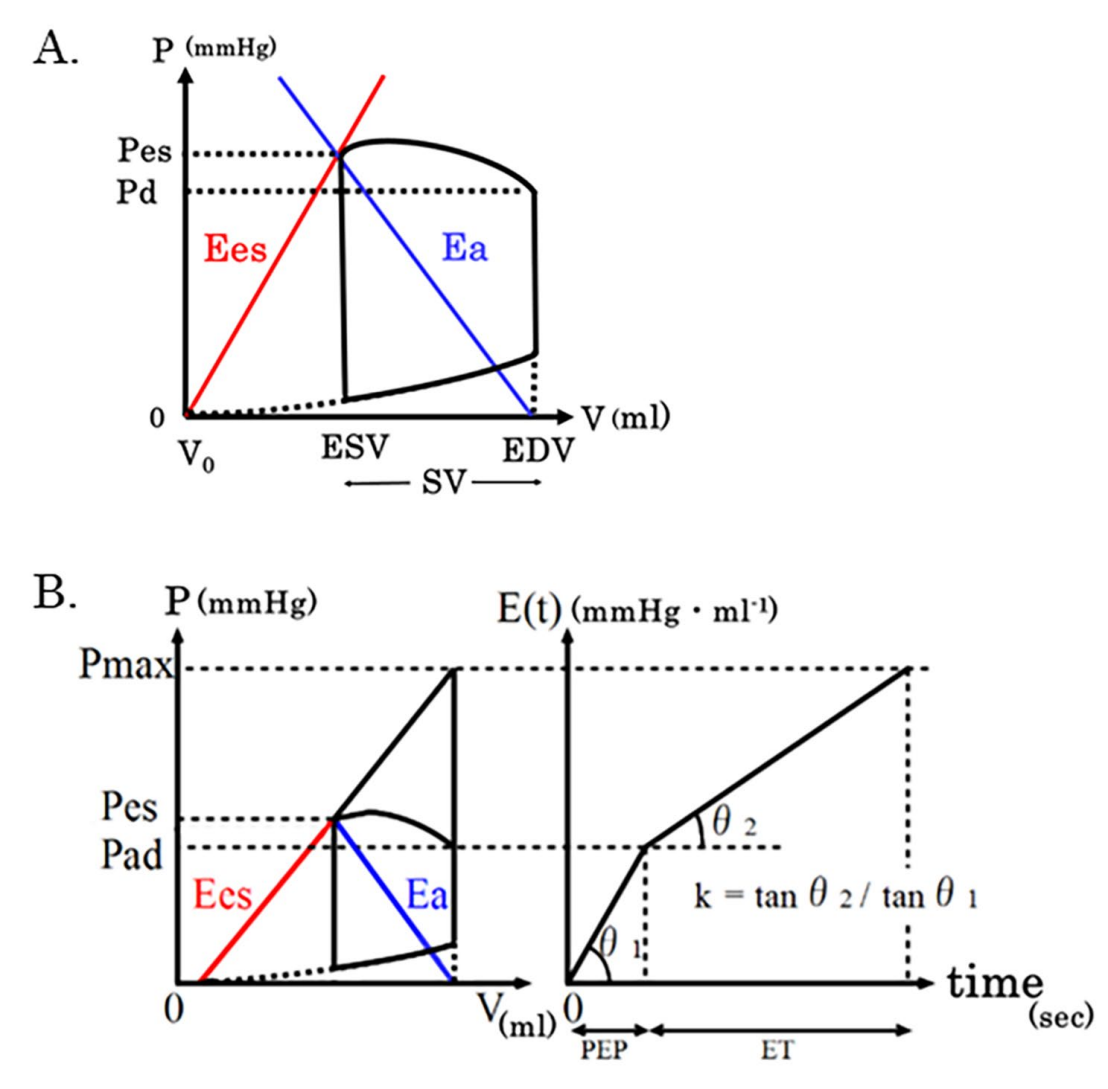
Cardio-Ankle Vascular Index and equation for estimating end diastolic volume (EDV). (**A**) Schematic drawing of a pressure-volume loop. PVA is the area obtained by adding A and (**B**) A: the approximately triangular area bound by the end systolic pressure volume relation, end diastolic pressure volume relation and left border of the pressure-volume loop. B: the area within the pressure-volume loop, expressed as EW. B. (Adapted from ref. 1) Schematic diagram of a pressure-volume loop. Ees: slope of the end-systolic pressure-volume relationship, Ea: slope of the line connecting the end-diastolic volume (EDV) on the volume axis to the end-systolic pressure volume point. Pes: end-systolic pressure, Pad: diastolic pressure, Pmax: putative isovolumic pressure, E(t): change in the elastance of the left ventricle on standing, k: ratio of the slope when E(t) was approximated bilinearly in the isovolumic contraction phase and isovolumic ejection phase, PEP: pre-ejection period, ET: ejection time

In a previous study, Hayashi et al. found that Ees/Ea can be estimated by a noninvasive method using four parameters, pre-ejection period (PEP), ejection time (ET), end-systolic pressure (Pes), and diastolic blood pressure (dBP) [5] (Fig. 1B). Since these parameters can be obtained using an electrocardiogram, pulse waveform and phonocardiogram, we adopted their method in this study to perform continuous estimation of Ees/Ea for every beat.

Here, E(t) was expressed as the change in elastance of the left ventricle on standing. The ratio of the slope when E(t) was approximated bilinearly in the isovolumic contraction phase and the isovolumic ejection phase was defined as k. The hypothetical Pmax that would develop if the aorta was clamped was expressed using PEP, ET, and Pad as:

2 $${\rm{Pmax}}\,{\rm{ = }}\,{\rm{Pad}}\,{\rm{ + }}\,{\rm{Pad}} \times {\rm{k}} \times {\rm{ET/PEP}}$$

In addition, when the increase in left ventricular pressure from Pes to Pmax and the increase in aortic pressure to Pes occur with the same ejection volume, the ratio of Ees to Ea can be expressed as

3 $${\rm{Ees/Ea}}\,{\rm{ = }}\,{\rm{(Pmax}} - {\rm{Pes)/Pes}}$$

Rearranging Eqs. 2 and 3,

4 $${\rm{Ees/Ea}}\,{\rm{ = }}\,{\rm{Pad/Pes}}\,{\rm{(1}}\,{\rm{ + }}\,{\rm{k}} \times {\rm{ET/PEP)}}\, - \,{\rm{1}}$$

Based on this theoretical formula and experiment,

5 $${\rm{k}}\,{\rm{ = }}\,{\rm{0}}{\rm{.53}} \times {{\rm{(Ees/Ea)}}^{{\rm{0}}{\rm{.51}}}}$$

Ees/Ea was calculated by simultaneously solving Eqs. 4 and 5 by Newton’s method.

Left ventricular efficiency (Eff) as a pump is defined as the ratio of external work (EW) to myocardial oxygen consumption, and strongly correlates with the pressure-volume area (PVA), as indicated by the pressure-volume loop [8] (Fig. 1A). In this loop, EW is the area within the pressure-volume loop, reflecting the amount of work performed by the left ventricle when pumping blood. Further, in the pressure-volume loop, the PVA is divided into two areas: one is the approximate triangular area bounded by the end-systolic pressure volume relation, end-diastolic pressure volume relation, and the left border of the pressure-volume loop (Fig. 1A); and the other is the EW. EW and PVA can be calculated as follows using the pressure-volume loop [9, 10].

6 $${\rm{Ees}}\,{\rm{ = }}\,{\rm{Pes/(ESV}} - {\rm{V0)}}$$

7 $${\rm{Ea}}\,{\rm{ = }}\,{\rm{Pes/SV}}$$

8 $${\rm{EW}}\,{\rm{ = }}\,{\rm{Pes}} \times {\rm{SV}}$$

9 $${\rm{PVA}}\,{\rm{ = }}\,{\rm{Pes}} \times {\rm{SV}}\,{\rm{ + }}\,{\rm{0}}{\rm{.5}} \times {\rm{Pes}} \times {\rm{(ESV}} - {\rm{V0)}}$$

10 $$\begin{array}{c}{\rm{Eff}}{\mkern 1mu} {\rm{ = }}{\mkern 1mu} {\rm{EW/PVA}}{\mkern 1mu} {\rm{ = }}{\mkern 1mu} ({\rm{Pes}} \times {\rm{SV}}){\rm{/}}\\({\rm{Pes}} \times {\rm{SV + 0}}.{\rm{5}} \times {\rm{Pes}} \times ({\rm{ESV}} - {\rm{V0}}))\end{array}$$

Rearranging Eqs. 6–10, gave us an equation to calculate Eff.

11 $${\rm{Eff}}\,{\rm{ = }}\,{\rm{1/(1 + 0}}{\rm{.5}} \times {\rm{Ea/Ees)}}$$

EF is the ratio of SV to left ventricular EDV.

12 $${\rm{EF}}\,{\rm{ = }}\,{\rm{SV/}}\,{\rm{EDV}}$$

Assuming V0 is zero, using Eqs. 10 and 12, EF (EFeff) can be approximated from Eff as:

13 $${\rm{EFeff }} \fallingdotseq {\rm{ Eff/(2}} - {\rm{Eff)}}$$

### Statistical analysis

SPSS was used for the statistical analysis. The sample size of subjects required in this study to produce an α = 0.05 and a power of 0.80 was adopted from a previous study [11]. The sample size was set at 45 patients, assuming a dropout rate of 20%.

EFecho and EFeff were compared using simple regression analysis and Pearson’s correlation analysis. Additionally, Bland-Altman analysis was used to assess agreement between the two [12]. EF measured by TTE (EFecho) was used as a reference. Bias (the mean difference between EFecho and EFeff), limits of agreement, and percentage error (PE) were calculated. The PE was calculated using the formula, PE = 2SD of the bias/mean value of reference methods. Continuous variables were reported as averages and standard deviations (SDs).

The agreement between EFecho and EFeff was interpreted as acceptable when the PE was ≤ 30% [13]. Significance was set at P < 0.05.

## Results

**Fig. 2:**
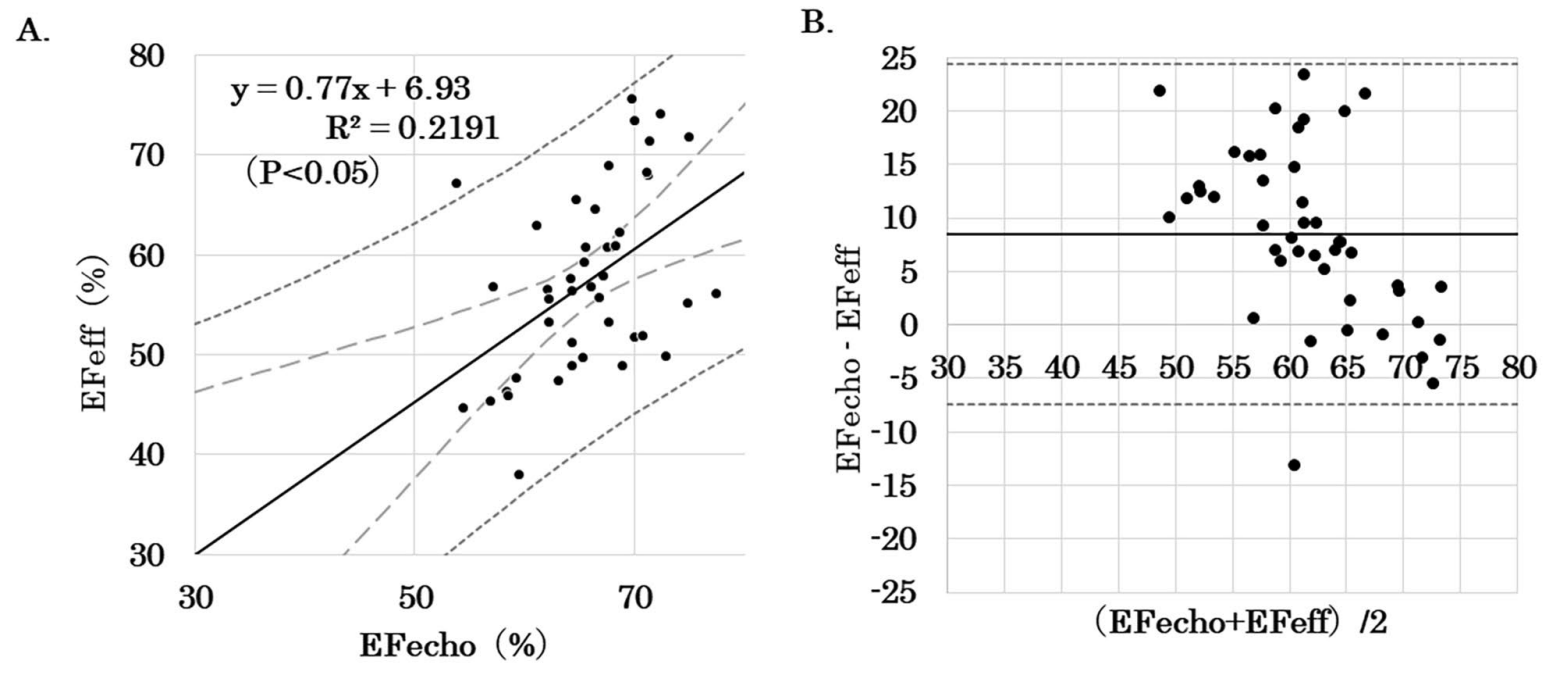
**A**: Correlation between EFecho and EFeff. The correlation coefficient (r) was 0.47. (R^2^ = 0.219, p < 0.05) **B**: Bland-Altman plot between EFecho and EFeff demonstrating a bias of 8.5 ± 8.0%. The solid line shows the bias and the dotted line represents ± 2SD.

The study included 44 healthy adults (36 males and 8 females). The characteristics of the participants are shown in Table 1. Their mean age was 24 ± 4 years, mean height was 169 ± 7 cm, and mean weight was 65 ± 11 kg. EFecho was 66 ± 5%, and EFeff was 57 ± 9%. There was a positive correlation between EFecho and EFeff (R^2^ = 0.219, P < 0.05) (Fig. 2A).

**Table 1:** Characteristics of the subjects (n = 44)

Characteristics	Value
Age, years	24 ± 4
Gender, female, %	18.2%
Height, cm	169 ± 7
Weight, kg	65 ± 11
BMI, kg/m^2^	23 ± 3
SBP,mmHg	119 ± 10
DBP,mmHg	72 ± 6
PEP,second	97 ± 14
ET,second	303 ± 18
Ees/Ea	1.5 ± 0.6
EF echo, %	66 ± 5
EF eff, %	57 ± 9

Data presented as mean ± standard deviation values, except where otherwise indicated. BMI; body mass index, SBP; Systolic blood pressure, DBP; Diastolic blood pressure, PEP; pre-ejection period, ET; ejection time, EF; ejection fraction

Bland-Altman analysis showed a bias of 8.5 ± 8.0%, with limits of agreement of − 7.5–24.4%. The PE was 24% (Fig. 2B).

## Discussion

In this study, EF was measured noninvasively using the left ventricular arterial coupling. The parameters of PEP and ET used in the present study were measured using a vascular screening system. Since Pes was measured by sBP and dBP using the formula published by Kappus et al. [6], EF was obtained completely without invasive examinations.

Comparative evaluation showed a correlation, compatibility and good agreement between EFeff and EFecho. Additionally, the PE of 24% indicates that the agreement between the two was clinically acceptable; thus, EFeff can be used as a substitute for EFecho. The EF of the normal heart ranges between 50% and 70% [14]. EF calculated using left ventricular arterial coupling in the present study was 57 ± 9%, which is consistent with standard criteria.

In a previous study [11], in which EF was calculated using two different methods, the limits of agreement were − 18.1–8.3%, Our study results indicated greater bias and variability between the two methods.

On the other hand, EF calculated using Ees/Ea is considered to have certain advantages over EF calculated using TTE (transthoracic echocardiography). It is difficult to calculate EF continuously from TTE. Additionally, TEE (transesophageal echocardiography) is highly invasive and might be associated with complications, such as odynophagia, upper gastrointestinal hemorrhage, and esophageal perforation [15]. It is, thus, difficult to use TEE for evaluating EF in all patients undergoing surgery. Our method enables continuous non-invasive monitoring of EF.

EF is expressed by the ratio of SV to Ved, as in Eq. 12, when V0 is assumed to be 0 ml. However, since the denominator is estimated to be lower than Ved, because, in fact, V0 is not 0 ml, EF calculated using TTE might be considered to be lower than the actual value. On the other hand, since Eff includes the influence of V0, Eff might reflect cardiac function more accurately.

In this study, CAVI was performed in the supine position, whereas TTE was performed in the left lateral position. This is because it is easier to apparatus the apex of the left ventricle in the supine position allowing more accurate calculation of EDV.

Although SV was measured by TTE in the present study, it is impossible to perform TTE continuously during surgery. Stroke volume and arterial pressure-based cardiac output, which is the cardiac output obtained using an arterial pressure waveform, can be obtained using a Flotrac hemodynamic monitoring system (Edwards Lifesciences, Irvine, CA, USA) [16]. Our future plan is to measure SV using such a device and to monitor EF and its changes continuously during general anesthesia.

There are several limitations in the present study. First, all of the study participants were young, healthy people with no cardiac diseases. On the other hand, since patients with cardiac disease occasionally have abnormal heart rhythms and might develop irregular Q waves, the values of PEP and ET cannot be accurately calculated. However, since the purpose of this study was to examine a method of calculating EF using left ventricular arterial coupling in persons with normal cardiac function, we decided to only include healthy participants in this study. In future, it will be necessary to examine whether it is possible to calculate EF using left ventricular arterial coupling in people with cardiac diseases, with similar correlation and agreement. Second, in this study, we assumed that V0 was 0 ml, which was only possible because the study included participants with normal cardiac function [17].

## Conclusion

In conclusion, the results of the present study suggest that EF can be monitored noninvasively and continuously using the parameter of left ventricular arterial coupling.

## Data Availability

The datasets used and/or analyzed during the current study are available from the corresponding author on reasonable request.
